# Contrasting functional structure of saproxylic beetle assemblages associated to different microhabitats

**DOI:** 10.1038/s41598-020-58408-6

**Published:** 2020-01-30

**Authors:** Estefanía Micó, Pablo Ramilo, Simon Thorn, Jörg Müller, Eduardo Galante, Carlos P. Carmona

**Affiliations:** 1 Centro Iberoamericano de la Biodiversidad (CIBIO), Universidad de Alicante, San Vicente del Raspeig s/n, 03690 Alicante, Spain; 2 Field Station Fabrikschleichach, Department of Animal Ecology and Tropical Biology, Biocenter University of Würzburg, Glashüttenstraße 5, DE‐96181 Rauhenebrach, Germany; 3 Bavarian Forest National Park, Freyunger Str. 2, 94481 Grafenau, Germany; 4 Institute of Ecology and Earth Sciences, University of Tartu, Lai 40, Tartu, 51005 Estonia

**Keywords:** Biodiversity, Community ecology

## Abstract

Saproxylic beetles depend on diverse microhabitats that are exploited by different species assemblages. We focused on anlyse the functional trait patterns and functional diversity components of two main assemblages that were collected with window traps (WTs) and hollow emergence traps (HETs) respectively, between three protected areas of the Iberian Peninsula. For that we measured phenological, physiological, morphological, and ecological traits. Results showed that the main microhabitats exploited by each assemblage (WT and HET) constrain most of the morphological traits and trophic guilds. In addition, relative elytra length and predator guild, together with adult activity period, responded to differences at the habitat level (among study areas). HET assemblages were less taxonomically diverse but more functionally diverse than those of WTs, enhancing the functional relevance of tree hollows. Additionally, niche filtering dominated WT assemblages, which were characterised by a narrower functional space and a higher redundancy. In contrast, in the HET assemblages the coexistence of functionally dissimilar species is driven by the niche heterogeneity. HET and WT assemblages differed in the functional space occupied by each within areas, but both assemblages reflected coincident patterns among areas that pointed to a reduction of functional space with management.

## Introduction

Saproxylic beetles are one of the main components of the forest fauna. They are highly diverse and provide important ecosystem services related to nutrient recycling and the decomposition of wood^1,2^; forest pest control, through the action of saproxylic predators on primary xylophagous beetles (i.e., Scolytinae)^3,4^; and pollination, as many adult saproxylic beetles are flower visitors. In spite of the functional importance of saproxylic beetle communities, few studies have included a functional trait approach^5–10^, and most have focused on how they respond to environmental change.

Saproxylic beetles depend, directly or indirectly, on a number of distinct microhabitats provided by living and dead trees that can host specific assemblages of co-occurring beetle species^1,11^. Moreover, each assemblage is formed by species belonging to different trophic guilds that interact among themselves and with the substrate in different ways^11,12^. Species richness and composition vary between woody microhabitats^6,13,14^; however, studies focused on the different saproxylic assemblages –understood as a taxonomically related group of species that occur together in space and time^15^– or on their assemblage mechanisms are scarce^6^.

The most commonly used method for studying saproxylic beetles is flight intercept traps, such as window traps (WTs), which collect a species assemblage representing a broad set of ecologies^6^. WTs primarily catch flying adults arriving from several tree microhabitats (e.g., decaying branches, bark, or tree hollows) but also from other woody resources within the woodland environment, such as dead wood on the ground (e.g., snags, logs)^16–18^. However, other methods, such as emergence traps (ETs), are more effective at capturing species linked, in different degrees, to specific microhabitats, such as tree hollows – considered ‘keystone’ structures in European forests^8^ – or logs, as they allow capturing saproxylic species shortly after their emergence from immature stages^13,18–20^.

The assemblages collected with emergence traps on specialised microhabitats, such as tree hollows or logs, differ in their species composition from those collected with window traps^6,13,21^. Differences in the species distribution patterns of WT and ET assemblages provide valuable information on the environmental variables affecting each. For example, assemblages collected with WTs reflect the forest structure, as they collect beetles that exploit different woody microhabitats. In comparison, tree hollow assemblages collected with an ET are influenced by the characteristics of each cavity^18^. In the same way, species trapped using emergence traps on logs (log assemblages) respond more weakly to environmental factors, i.e., to deadwood succession, than those trapped using flight intercept traps^6^. Yet, approaches that only use such taxonomic data restrict the predictive power of community studies^22–24^, while a trait approach can reflect important differences between species such as the ways that they use resources, respond to local environmental change and influence their environment^24–27^. Moreover, the information provided by functional traits may help to understand the assembly processes^25,26,28–30^. Here we follow Götzenberger *et al*.^31^ in considering assembly rules as any constraint on species coexistence. In particular, we focus on those associated to ecological filters such as dispersal, the abiotic environment, and biotic interactions.

Despite being one of the main goals of ecology, understanding the assembly rules (e.g., environmental filtering, competition, predation, etc.) has been very underexplored in saproxylic beetle assemblages^12^. In this regard, there is still a need to lay the foundations for understanding saproxylic beetle functional diversity, which could shed light on questions related to their assembly rules and the effects of that diversity on ecosystem functioning. Taking a further step in the study of saproxylic beetle functional diversity implies the following: 1) to expand the number and types of functional traits that are assessed; until now, the most commonly used functional traits in saproxylic beetle studies have been trophic guild, body size and microhabitat preferences and 2) to deepen our understanding of the trait structure of the main saproxylic assemblages (e.g., tree hollows, logs, WT assemblages) and how their diversity patterns differ.

Within this framework, our main aim was to analyse the functional trait diversity of tree hollow and window trap assemblages (henceforth HET and WT assemblages respectively) using different kinds of functional traits, including morphological, ecological (trophic guilds), phenological and a subrogate of physiological traits. We hypothesise that the microhabitats exploited by each assemblage will determine their functional space and their assembly processes. Mature *Quercus pyrenaica* forests in three protected areas in the Mediterranean region of the Iberian Peninsula were selected to test the following predictions derived from this hypothesis:

- The selected functional traits will show differences at both microhabitat level (between HET and WT assemblages), and at habitat level (between study areas).
- The higher taxonomic richness should not necessarily imply a greater functional space. HET assemblages are expected to exhibit a broader functional space and lower redundancy due to niche heterogeneity within each tree hollow^32^.
- HET and WT assemblages are expected to differ in their main assembly rules and in the functional space occupied because the kind and heterogeneity of microhabitats exploited by each assemblage differ^12^.

## Results

A total of 12,394 individuals (3,885 in HETs and 8,509 in WTs) belonging to 346 saproxylic species (147 in HETs and 322 in WTs) and 47 families (34 in HETs and 45 in WTs) were identified (See Supplementary Table S1).

Inventory completeness of HET and WT assemblages was 99.1 and 99.0% respectively. Likewise, the inventory completeness values for each assemblage at each study area were also consistently very high (>97.2%). After omitting rare species (singletons and doubletons, 130 species) and those with more than 4 traits lacking (16 spp.) (see data analysis), the analysis focused on 12,025 individuals of 200 species across 35 families (See Supplementary Table S1). The numbers of species and individuals collected with emergence and window traps in each area are shown in Supplementary Table S2.

### Trait patterns

**Figure 1:**
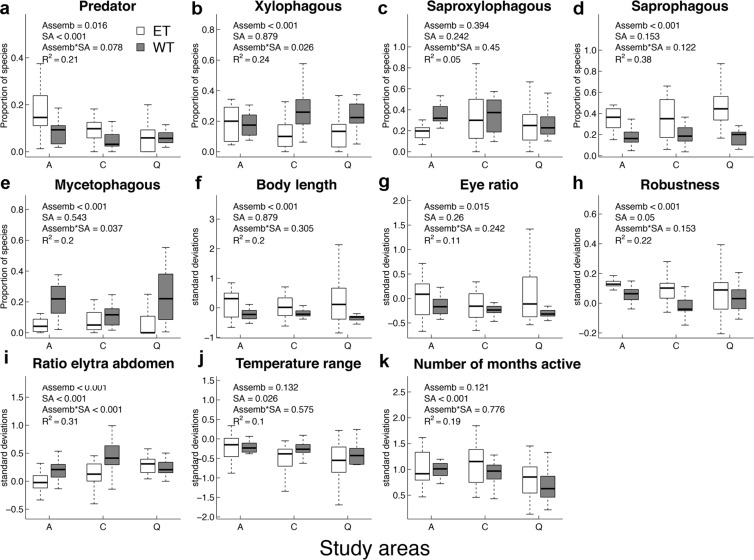
Effects of the kind of assemblage (Assemb; hollow assemblage (ET) (in white) or collected by window trap (WT) (in gray)), the study area (SA; A: Azaba, C: Cabañeros, Q: Quilamas) and its interaction on the community weighted mean values of the different traits considered (**a**,**b**). P values associated to each factor were obtained by linear regression models and adjusted to control for false discovery rate.

Regarding morphological traits, body length and robustness were greater in HET assemblages than in WT assemblages, while no difference was found among areas (Fig. 1f,h). In contrast, relative elytra length showed differences among both assemblages and areas, being higher in window trap assemblages and in Cabañeros (Fig. 1i).

Moreover, trophic guilds exhibited different compositional patterns between assemblages. In this way, while xylophagous and mycetophagous species were more abundant in WT assemblages, HET assemblages were dominated by saprophagous species (Fig. 1b,d,e). Predators species were the only guild that showed differences among areas, the “dehesa” ecosystem (Azaba) being the richest one (Fig. 1a). No difference in saproxylophagous abundance was found between assemblages or among areas (Fig. 1c).

The number of months in which the species were active differed between areas. In contrast, the range of temperatures of the months in which adults were active (physiological trait) did not differ significantly between assemblages or among areas.

### Taxonomic richness and functional diversity

**Figure 2:**
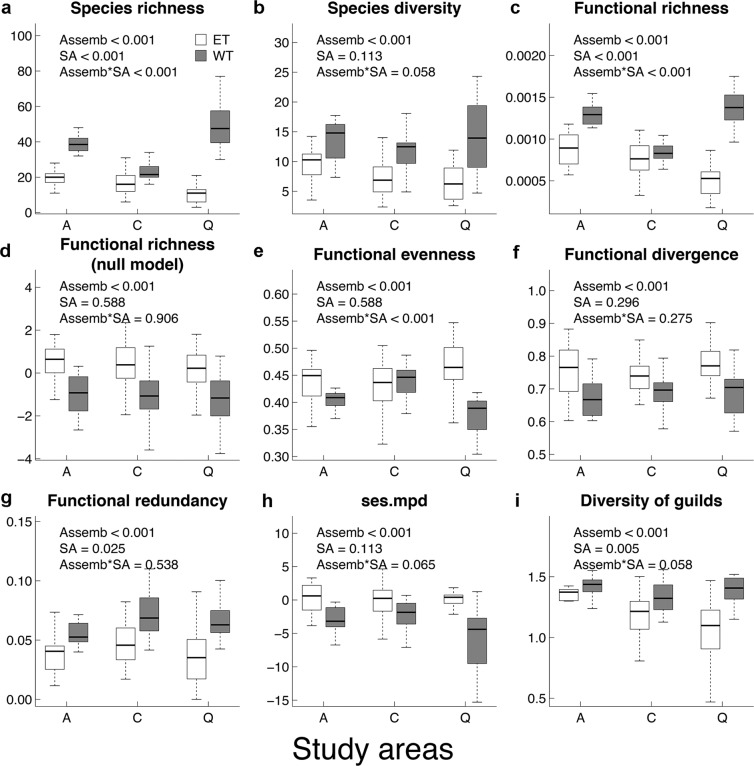
Effects of the kind of assemblage (Assemb; hollow assemblage (ET) (in white) or collected by window trap (WT) (in gray)), the study area (SA; A: Azaba, C: Cabañeros, Q: Quilamas) and its interaction on the different indicators of the taxonomical (**a**,**b**) and functional diversity (c-i) (considering all traits simultaneously, see main text for further details) P values associated to each factor were obtained by linear regression models and adjusted to control for false discovery rate.

Species richness varied between assemblages and among areas, always being higher in WT assemblages than in tree HET assemblage (Fig. 2a). The same pattern was obtained using the species diversity (equivalent number of species), although differences among areas ceased to be significant (Fig. 2b). The removal of singletons and doubletons did not affect either of those metrics (See methods and Supplementary Fig. S3).

There was a strong interaction between the type of assemblage and study area, as Quilamas was the richest forest for WT assemblages and the poorest for HETs (Fig. 2a). Functional richness followed the same pattern as species richness (Fig. 2c), with a very high positive correlation between these two variables (r = 0.95).

However, with SES-functional richness, the result was inverted: HET assemblages exhibited a greater functional volume than WT, and differences among areas were not significant (Fig. 2d).

Both FEve and FDiv showed similar patterns, being higher for tree hollow assemblages. Although there were no differences between study areas, the two assemblages of Quilamas were again markedly different for these indicators (significant assemblage: area interaction; Fig. 2e,f).

We also found differences between assemblages in their functional redundancy, which was higher in the WT than in HET assemblages, again with some differences between study areas (Fig. 2g). Finally, the average dissimilarity between species, indicated by MPD, was lower in the WT assemblages than in the HET assemblages (Fig. 2h).

### Taxonomic and functional composition

**Figure 3:**
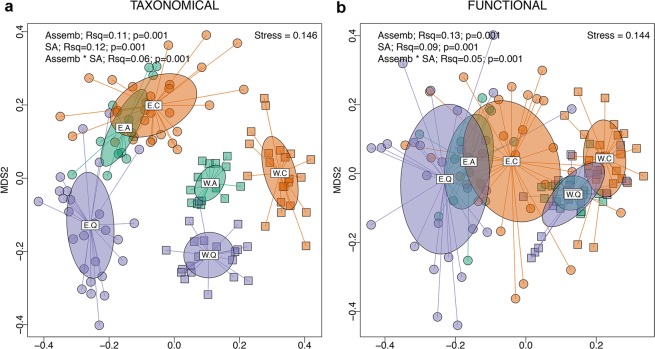
Taxonomical (**a**) and functional (**b**) composition of communities classifed according to the kind of assemblage they belong to (Assemb; hollow assemblage or collected by window trap, E and W, respectively), and the study area (SA; A: Azaba, C: Cabañeros, Q: Quilamas). Each point represents a community in a NMDS space based on the taxonomical and functional dissimilarities between communities. Each plot shows the results of a PERMANOVA analysis (999 permutations), including the proportion of explained variance by each explanatory variable (Rsq) and its significance level.

The assemblage type, WT versus HET, was a more important driver of the compositional differences between samples than the study area at the functional level (Fig. 3b), and they were similarly important at the taxonomical level (Fig. 3a). Both assemblages were clearly differentiated at both the functional and taxonomical levels, as revealed by NMDS. On the other hand, differences among areas were higher for WT assemblages than for HET ones (Fig. 3a,b). Although the functional composition was less differentiated than the taxonomic composition, both assemblages remained differentiated. Furthermore, differences between samples within study areas were in general larger for HET assemblages than for WT ones, both at the taxonomic (PERMDISP test: average difference to median in HET = 0.59; difference to median in WT = 0.53; p < 0.001; Fig. 3a) and the functional level (PERMDISP test: average difference to median in HET = 0.51; difference to median in WT = 0.40; p<0.001; Fig. 3b). Although functional turnover within areas was clearly higher in hollows, both assemblages exhibited the same pattern of dissimilarity among areas, where the Quilamas and Azaba areas showed greater and lesser functional turnover, respectively (Fig. 3b).

## Discussion

This paper analyses the patterns of functional trait composition and diversity of two assemblages that depend on different microhabitats (WTs assemblage and hollow emergence traps) between three protected areas in the Iberian Peninsula. This approach has revealed that both assemblages differed in their assembly rules and in their patterns of occupation of the functional space within areas. However, management had similar effects on both assemblages, reflected by the effect of study area. In addition, we expand the number and type of traits normally used in saproxylic beetle studies and show that different type traits were informative at different levels (microhabitat and habitat level).

The analysis of the functional structures of two different assemblages (HET assemblage and WT assemblage) in three protected areas showed that functional richness was positively correlated with species richness, and in all cases, differences among assemblages and areas were observed. Functional richness values were thus higher in WT assemblages, in which both the number of species and the equivalent number of species were higher. It is not uncommon to find that increasing species diversity lead to increasing functional diversity, as the functional characteristics of coexisting organisms must differ at some level^33,34^. After removing the effect of species richness on FRic throughout a null model, the aforementioned trend was reversed. HET assemblages exhibited a greater functional diversity, and differences among areas ceased to be significant (Fig. 2d). Note that higher functional diversity in tree hollows in comparison with other deadwood microhabitats in beech forests was also pointed by Müller *et al*.^8^ using null models. This means that WT assemblages were more functionally redundant, which was also in agreement with the information provided by the functional redundancy and MPD indices (Fig. 2g,h). Furthermore, the HET assemblages showed higher evenness, revealing a higher regularity of the distribution of abundance in the functional space. They also showed greater functional divergence, which means that the most abundant species are very dissimilar. This result suggests that competitive interactions between species in hollows are likely to be weak^33^.

The high levels of functional divergence in tree hollows could be associated with a high degree of niche differentiation among species. Limiting similarity principles^35^ or competitive exclusion^36^ might be driving tree hollow assembly processes, thus dampening niche filtering in this case. In this regard, Sánchez-Galván *et al*.^12^ studied non-random co-occurrence patterns of saproxylic beetles and Syrphidae (Diptera) in 72 tree hollows and concluded that species interactions (i.e., predation and facilitation), together with habitat segregation, were the main factors shaping tree-hollow assemblages in Mediterranean *Quercus* forests, while competition seemed to be less important. This observation is consistent with the notion of tree hollows as heterogeneous multi-habitat systems simultaneously housing a wide range of microhabitats^32,37^. Our results, in agreement with Sánchez-Galván *et al*.^12^, suggest that the niche heterogeneity within each hollow favours the stable coexistence of functionally dissimilar species in tree hollow assemblages.

In all cases, FRic null model values from the WT assemblages were lower than expected by chance, suggesting that niche filtering could be acting in this assemblage^38^ (Fig. 2d). Unlike HET assemblages, the WT assemblages combine species from a variety of microhabitats related to the tree (e.g., decaying branches, bark, and tree hollows) as well as other resources in the surroundings, such as dead wood on the ground (e.g., snags, logs). The exploitation of these confined microhabitats, mainly wood and bark, might be filtering functional traits toward some kind of functional syndrome. Overall, most morphological traits showed different patterns between assemblages but not among areas. Body length and robustness were lower in WT assemblages than in HET assemblages, which allows us to hypothesise the existence of a size filter in the WT assemblage (Fig. 1f,h).

The only morphological trait that showed changes between assemblages and areas was the relative length of the elytra. For carabid beetles, short elytra indicate better dispersal efficiency^24,39,40^, while longer elytra provide wings with protection, which allows access to rugose habitats without damaging the wings^41^. In our case, the relative length of the elytra also differed between assemblages, being higher in WT assemblages. Again, this reflects a predominance of species from more confined microhabitats (i.e. inside wood, under bark) in which long elytra protect wings instead of reflecting a bias to a better dispersal efficiency. However, differences in elytra length were also found among areas. Although the inverse relationship between elytra length and dispersal ability has yet to be tested empirically^27^, the higher number of species with greater dispersal abilities in Azaba could be interpreted as a response to disturbance, as this was the most managed of the three areas. Azaba is a typical “dehesa” characterised by the presence of scattered trees in open woodland, in which traditional management affects both landscape and tree structure. However, more studies are needed to confirm this hypothesis.

The types and characteristics of microhabitats exploited by each assemblage not only constrain morphological traits, they also affect trophic guild distributions. In this way, although trophic guild diversity was greater in WT assemblages, each guild exhibited different abundance patterns between the assemblages (Fig. 1a-e). The dominance of xylophagous and mycetophagous species in the WT assemblage is not surprising if we consider that the bark and wood of living trees and of standing and fallen deadwood are the main microhabitats exploited by these species. Nevertheless, the most important substrate of the cavities is normally a wood mould composed of borings, excrement, and carcasses, whose volume and quality can vary greatly among different cavities^1,42–45^. In this regard, wood mould quality is favouring saprophagous species in tree hollows (Micó *et al*.^44^). In contrast, predator abundance differed between areas, the tree hollows of the “dehesa” ecosystem (Azaba) being the richest in predators (Fig. 1a). In the “dehesa” ecosystem (the most managed area), the higher abundance of large and horizontal tree hollows containing larger volumes of wood mould^37,46,47^ could be favouring substrate-dependent guilds such as predators and saprophagous. Moreover, the degree of polyphagy of predators is likely to influence their response to changes in habitat landscape structure^48^. For example, some generalist spiders appear to exhibit preferences for agricultural over less-disturbed habitat^49^.

The phenological trait (number of months in which the species were active) differed only among areas, with the average values being higher in the southern area (Cabañeros) for both assemblages. In contrast, the physiological trait (temperature range of the months in which the species were active) did not differ among areas or assemblages. Taking into account that in ectotherm animals activity time constrains species distribution and their ability to respond to environment changes^50^, we should expect a lower impact of climatic change for assemblages with greater adult activity time. However, the usefulness of this trait for predicting the response of saproxylic assemblages to climate change in Mediterranean areas should be tested.

Regarding dissimilarity between assemblages and distributional patterns, our results showed that different assemblages from the same area differed more than the same assemblage from different areas in both species and functional trait composition (Fig. 3). Distinct saproxylic microhabitats harbour assemblages that vary in species richness and composition^51,52^. Differences in the studied assemblage can also lead to significant differences in the identified patterns of species distribution and turnover^18^. However, studies of functional turnover are far less numerous in spite of their potential to infer the mechanisms underlying community assembly and dynamics^53^. Both taxonomic and functional turnover within areas was always higher for HET assemblages (Fig. 3). This result probably reflects the fact that each cavity represents a very unique and complex environment, housing a wide range of microhabitats. As a consequence of this heterogeneity, there is high species and functional turnover among hollows, even within the same area^18,20,54^. However, regarding turnover among forests (Fig. 3), while species turnover was clearly greater for the WT assemblage, as they depend on different microhabitats influenced by forest structuring, functional turnover showed similar patterns of dissimilarity for both assemblages, as trait composition does not necessarily depend on taxonomic composition. For example, for both assemblages, Quilamas showed greater functional turnover among traps and totally overlapped those from Azaba, while both shared a smaller portion of the functional space with traps from Cabañeros. In addition, the most intensively managed area, Azaba, showed the lowest functional turnover among traps within each assemblage, likely suggesting some kind of homogenisation resulting from tree and forest landscape management^55^. This pattern has occurred with ant assemblages in managed forests^56^. Meanwhile, Cabañeros, the most natural area, did not show signs of ancient management, and Quilamas presents an intermediate situation.

We conclude that the functional trait approach was able to provide important structural properties of the different saproxylic assemblages and to infer the assembly mechanisms. In this way, while WT assemblages seem to be more diverse on the basis of species richness and diversity, a trait approach revealed that they were also more functionally redundant. In contrast, hollow ET assemblages showed greater functional diversity, enhancing the importance of tree hollows as key habitats for the maintenance of functional diversity in Mediterranean forest ecosystems.

Moreover, this trait approach was able to better reflect coincident patterns among areas than a taxonomic approach by providing extra information. Specifically, a strong reduction of the functional space in both assemblages was detected with forest management. This study demonstrates the usefulness of functional trait approach to understand the assemblage mechanisms and diversity patterns of saproxylic beetles at microhabitat and habitat level and encourages to depeen in the effect of environmental changes on saproxylic functions.

## Materials and Methods

### Study area

Fieldwork was conducted in Mediterranean oak forests dominated by *Quercus pyrenaica* Willd. located in three protected areas of the Iberian Peninsula (Supplementary Fig. S4).

Cabañeros National Park (henceforth Cabañeros) is located in central Spain (39° 239′ 470′′ N, 4° 299′ 140′′ W). With altitudes between 560 and 1448 m, the park features 40,856 ha of well-preserved Mediterranean ecosystems, with few signs of management, including several patches of forest dominated by *Quercus* species (^57,58^). Mixed forest of *Q. pyrenaica* and *Q. faginea* Lam. stands is scattered throughout the park, primarily in valleys, with a total extent of 634 ha^18^.

Campanarios de Azaba Biological Reserve (henceforth Azaba) is located in western Spain (40° 29′ 60′′ N, 6° 46′ 50′′ W). It is a private reserve of 522 ha at an altitude of 800 m. The landscape is a typical ‘dehesa’, characterised by the presence of scattered trees in open woodland dominated by deciduous forest of *Q. pyrenaica* and *Q. faginea* and evergreen forests of *Q. rotundifolia* Lam^18,59^. Tree management in this area has consisted of pollarding, which involves the suppression of the main branch of the tree^59^. Pollarding practices result in the formation of many scars and large, horizontal cavities in the trunk of the trees^59^.

Sierra de las Quilamas Natural Area (henceforth Quilamas) is located in western Spain (40° 30′ 10′′N, 6° 05′ 15′′ W). The area is 11,100 ha and ranges in altitude between 600 and 1,400 m. *Q. pyrenaica* is the dominant tree species^18^. Signs of ancient pollarding activity are noticeable on trees in this area, but currently, no active management is occurring.

### Sampling methods and species identification

Two saproxylic beetle assemblages were sampled: a tree hollow assemblage, collected with ETs (henceforth HET assemblage), and a window trap assemblage (henceforth WT assemblage). The WT assemblage is associated with a variety of tree microhabitats as well as other woody resources within the woodland environment (see Introduction Section). Adult saproxylic beetles were collected using hollow emergence traps (ETs) and window traps (WTs), all of which were placed on deciduous *Quercus* species (*Q. pyrenaica* and *Q. faginea*).

Each ET consisted of a black acrylic mesh that completely seals the tree hollow and a catcher pot attached to the mesh^13,19^. Each WT consisted of two cross -transparent sheets lying over a funnel and a collection container^60^. WTs were hung from live trees 1.5–2 m above the ground. All the selected woodlands have a tree trunk separation **>**3 m between them, which increases the efficiency of this kind of trap for studying the saproxylic beetles associated with each tree and its sourrondings^58^. In both types of traps, ethylene glycol or propylene glycol 70% was used as an odourless preservative that avoid any influence on the catches. A total of 69 ETs and 56 WTs were distributed across the three selected areas (Table 1, Supplementary Fig. S4). In each area, traps were checked monthly for 12 consecutive months avoiding differences among areas due to season effect (Table 1).

**Table 1:** Number of traps and sampling period in each one of the three selected locations Quilamas, Sierra de las Quilamas Natural Area; Cabañeros, Cabañeros National Park; Azaba, Biological Reserve ‘Campanarios de Azaba’.

Site	Hollow Emergence Traps		Window Traps	
	N°	Sampling period	N°	Sampling period
Cabañeros	29	Apr 2009-Mar 2010	22	Sep 2004-Aug 2005
Quilamas	27	May 2012-Apr 2013	20	May 2012-Apr 2013/ Apr 2014- Mar 2015
Azaba	13	May 2010-Apr 2011	14	May 2010-Apr 2011

Nomenclature is according to Fauna Europaea (http://www.faunaeur.org/), Bouchard *et al*.^61^, and the Catalogue of Palaearctic Coleoptera^62–66^. We had the support of specialists in different beetle families for species identification (see ‘Acknowledgments’ section). The specimens are deposited in the entomological collection of the University of Alicante (Collection CEUA) at CIBIO (Spain).

### Trait selection and measurement

Four different kinds of functional traits—morphological (M), ecological (E), phenological (Ph) and physiological (P)—were chosen to characterise both hollow and window trap assemblages (Table 2). For morphological traits, one to ten specimens of each species (depending on availability) were first photographed. Photos were produced as stalks of individual images made with a camera (Leica DFC 450) attached to a binocular stereomicroscope (Leica M205 C) allowing us to have the specimen all in focus. The measurements were then made on the photo using Leica Application Suite (LAS) software version 4.6.1. Some traits such as body size, depth and eye surface required a photo in lateral view while those of width required a dorsal image (see Table 2 for measurement details and units).

**Table 2:** Morphological traits used to calculate functional diversity and its functional significance for saproxylic beetle assemblages sampled in the three selected locations. Measurement details in first column.

*Trait*	*Functional significance*
**Body length** (M, Quantitative) Total lateral length from anterior of head to the apex of abdomen (expressed in mm).	Body size has been used as a predictor of microhabitat^24^ and as a response trait to understand community responses to changes in the environment (see^27,85^). Moreover, it has been used as a useful effect trait for different trophic guilds. For example, large coprophagous beetles had a higher impact on ecosystem functions such as dung removal and seed dispersal than small species^86^.
**Robustness** (M, Quantitative) was calculated by regressing five individual measures (pronotum dorsal maximum width and length and lateral depth; elytra maximum dorsal width and head maximum dorsal width) on body length and averaging the residuals of these regressions for each species^24^. Accordingly, a “robust” species will have large residuals, implying higher values in these variables than expected for its body length, making this trait effectively independent of body length.	Relative robustness is correlated with microhabitat use for beetles^24^. For instance, flattened body shapes are more common in species living in confined microhabitats, whereas more rounded shapes are found in open microhabitats^24^.
**Ratio Elytra length** (M, Quantitative) Ratio between maximum lateral length of elytra and maximum lateral length from the base of elytra to the apex of abdomen (expressed in mm).	In carabid beetles, the ratio of elytra length to abdomen length is connected to flight ability, as short elytra indicate better dispersal efficiency^24,39,40^. In contrast, long elytra can also act as a microhabitat predictor because they provide wing protection that allows access to rugged habitats without wing damage^41^.
**Eye size** (M, Quantitative) we measured the relative eye size as the ratio of the eye surface and head width. Eye surface was the area contained in a line depicting the eye perimeter (expressed in mm^2^).	This sensory trait can vary between trophic or taxonomic groups^27^ and may indicate the microhabitat use and structure and the lifestyle of species^87,88^.
**Trophic guild** (E, Qualitative) The assignment of the different categories—predatory, xylophagous, saproxylophagous, saprophagous, mycetophagous—was done at the species level based on literature, the FrisBE database^89^, Audisio *et al*.^90^, and the advice of the specialist of each family (pers. comm.).	Feeding guilds can be used as both effect and response traits that can be linked to resource use in each assemblage (i.e., feeding guilds have been used to show the impact of habitat fragmentation^91,92^.
**Number of months active** (Ph, Qualitative) Number of months that each species was active throughout the year.	Together with ecological performance traits and physiology, these types of traits are considered key predictors of extinction risk due to climate change^93^.
**Temperature range** (P, Quantitative) was calculated as the difference between the maximum and minimum temperature of the months in which adults of each species were active. Temperature was measured throughout the whole year of sampling with Temperature/Relative Humidity Data Loggers placed in the areas where the traps were located.	This trait can be considered a proxy of the physiological thermal range of the species and could be useful for predicting some of the consequences of climate change for species spatial distributions^47^. In this way, one species active for several months in only one area could show the same thermal range as another species active for only one month but distributed in several areas.

### Data analysis

Full inventory completeness was reported as the percentage of observed species in relation to the number of species predicted by the sample coverage estimator suggested by Chao and Jost^67^. Completeness was also evaluated for each sampling method and each sampling area. The analysis was performed with SPADE software^68^.

For the rest of the analysis, singletons and doubletons were removed from the data matrix because more than 2 specimens were needed to measure the phenological and physiological traits. The potential usefulness of both kinds of traits in studies related to the functional diversity of terrestrial beetles has been suggested by Fountain-Jones^27,69^. Moreover, we also removed the species for which information was lacking for more than 4 traits.

Two metrics of taxonomic diversity were calculated for each assemblage in every study area. We used species richness and the equivalent number of species (species diversity) as the inverse of the Gini-Simpson index, as in Pavoine *et al*.^70^.

For functional analysis, quantitative traits were log-transformed and scaled to 0 mean and unit variance. Then, using the abundance of each species in each sample, we estimated the community-weighted mean (CWM^71^) of each trait, which indicates the trait values of the most abundant species in the assemblage. We then estimated Gower’s dissimilarity matrix between all pairs of species and used it to perform a Principal Coordinates Analysis (PCoA). This strategy allows the combination of categorical and continuous variables and reduces trait dimensionality^72,73^. We retained the four first axes of the PCoA, that represented 65.6% of the total variation in the trait matrix, and used the scores of the species in these axes to estimate functional diversity indices. For this, we used the R package TPD^74,75^ to obtain a trait probability density function (TPD) for each species and assemblage. TPD functions reflect the abundance of the different trait combinations in each community and can be used to estimate several aspects of functional diversity^56,72^. Specifically, we estimated three components of functional diversity: functional richness (FRic, an indicator of the amount of functional space occupied by an assemblage); evenness (FEve, which reflects the uniformity of the distribution of abundance within the trait space) and divergence (FDiv, which reflects the degree to which the distribution of abundance of traits is concentrated close to the centre of the distribution^72,76^.

Functional richness is not independent of species richness^77^. Hence, to remove spurious effects of species richness on FRic values, we compared observed FRic values to a null model generated by means of a matrix-swap null model based on the species presence-absence matrix^78^. We restricted permutations so that only species coexisting in the same study area were swapped. We performed 500 randomisations and estimated the standardised effect size (SESFRic = [observed FRic – (mean of simulated FRic)/(SD of simulated FRic)]), which is an indicator of functional richness independent of species richness^79^. In addition, we estimated the functional redundancy of the species in the different assemblages^72^. An assemblage with a high degree of redundancy indicates that on average, the removal of a random species should not highly affect its functional structure, whereas when a species is removed from a community with low functional redundancy, it is more likely that the community functional structure will be modified^80^. Since functional redundancy is also not independent of species richness, we divided it by its upper bound (species richness – 1) in order to obtain relative values that are species-richness independent^72^. We also estimated the mean pairwise distance (MPD^38^) between species in each assemblage. High MPD values reflect that the coexisting species in an assemblage are functionally quite different, whereas low values indicate that most of the species in the assemblage have similar functional traits. Although MPD is not trivially related to species richness^38^, its variance decreases as species richness increases. We corrected this by performing the same type of null model explained above for FRic, so that we estimated SESMPD values.

Finally, we estimated the diversity of trophic guilds within each sample by means of the Shannon index of diversity, considering the relative abundance of each of the five groups in this trait.

We used the above-described indicators of taxonomic (species richness) and functional (FRic, FEve, FDiv, functional redundancy, and MPD) diversity as response variables in linear models (except in the case of species richness in which we used a generalized linear model with Poisson distribution), where the type of assemblage (HET or WT), the study area and the interaction of those two variables were used as predictors. We adjusted p-values to control for false discovery rate^81^.

In addition, we explored the effects of assemblages and study area on the taxonomical and functional turnover between assemblages. We calculated the taxonomical dissimilarity between each pair of areas using Bray-Curtis distances and their functional dissimilarities using overlap-based analysis of their TPD functions^82^. We analysed the resulting dissimilarity matrix using PERMANOVA (R package vegan^83^), using the kind of assemblage, the study area and its interaction as explanatory variables. The same dissimilarity matrix was used to estimate the distance of each combination of assemblage and study area to the centre of its class centroid (PERMDISP^83^). We finally performed Nonmetric Multidimensional Scaling (NMDS) analysis for both the taxonomical and functional pairwise areas dissimilarity matrices to help in visualising these analyses^84^.

## Supplementary information

Supplementary Table S1 and S2 and Figure S3 and S4.

## Data Availability

The datasets generated and analyzed during the current study are available in Figshare repository.
