# Author Correction: Contrasting functional structure of saproxylic beetle assemblages associated to different microhabitats

**DOI:** 10.1038/s41598-020-63411-y

**Published:** 2020-04-27

**Authors:** Estefanía Micó, Pablo Ramilo, Simon Thorn, Jörg Müller, Eduardo Galante, Carlos P. Carmona

**Affiliations:** 1 Centro Iberoamericano de la Biodiversidad (CIBIO), Universidad de Alicante, San Vicente del Raspeig s/n, 03690 Alicante, Spain; 2 Field Station Fabrikschleichach, Department of Animal Ecology and Tropical Biology, Biocenter University of Würzburg, Glashüttenstraße 5, DE-96181 Rauhenebrach, Germany; 3 Bavarian Forest National Park, Freyunger Str. 2, 94481 Grafenau, Germany; 4 Institute of Ecology and Earth Sciences, University of Tartu, Lai 40, Tartu, 51005 Estonia

Correction to: *Scientific Reports* 10.1038/s41598-020-58408-6, published online 30 January 2020

The original version of this Article contained a typographical error in the Abstract.

“We focused on anlyse the functional trait patterns and functional diversity components of two main assemblages that were collected with window traps (WTs) and hollow emergence traps (HETs) respectively, between three protected areas of the Iberian Peninsula.”

now reads:

“We focused on analysing the functional trait patterns and functional diversity components of two main assemblages that were collected with window traps (WTs) and hollow emergence traps (HETs) respectively, between three protected areas of the Iberian Peninsula.”

This has now been corrected in the PDF and HTML versions of the Article.

