# Supplementary material for: Contrasting functional structure of saproxylic beetle assemblages associated to different microhabitats
Source: Sci Rep. 2020 Jan 30;10:1520. doi: 10.1038/s41598-020-58408-6 (PMC6992628; doi:10.1038/s41598-020-58408-6)

Contrasting functional structure of saproxylic beetle assemblages associated to different microhabitats. Estefanía Micó, Pablo Ramilo, Simon Thorn, Jörg Müller, Eduardo Galante, Carlos P Carmona

Table S1
Species and number of individuals of saproxylic beetles collected. *Rare species (singletons and doubletons), **species with information available for less than 4 traits

Family	Species	Individuals
Aderidae	*Aderus populneus*	36
	*Euglenes oculatus***	4
	Otolelus neglectus	9
Anthribidae	*Allandrus therondi*	3
Biphyllidae	*Diplocoelus fagi*	13
Bostrichidae	*Lichenophanes numida**	2
	*Sinoxylon perforans**	1
	*Sinoxylon sexdentatum**	1
	Xylopertha praeusta	5
	*Xylopertha retusa**	2
Bothrideridae	*Bothrideres interstitialis*	6
	Oxylaemus cylindricus	13
Brentidae	*Amorphocephala coronata*	25
Buprestidae	*Acmaeodera degener*	10
	*Acmaeodera nigellata**	1
	Acmaeoderella adspersula	9
	*Acmaeoderella discoidea***	14
	Acmaeoderella flavofasciata	5
	Acmaeoderella moroderi	4
	*Agrilus angustulus**	1
	*Agrilus biguttatus**	2
	*Agrilus curtulus**	1
	*Agrilus elegans**	2
	*Agrilus hastulifer**	2
	*Agrilus laticornis**	1
	Anthaxia carmen	9
	Anthaxia millefolii	16
	*Anthaxia parallela**	1
	Anthaxia salicis	5
	*Anthaxia sepulchralis**	1
	Chrysobothris affinis	36
	*Eurythyrea quercus**	2
Cantharidae	*Malthodes forcipipher*	16
Carabidae	*Dromius agilis*	6
	Lamprias rufipes	10
	Lebia marginata	3
	*Lebia trimaculata**	2
	Metadromius myrmidon	3

Family	Species	Individuals
	*Philorhizus melanocephalus**	1
	*Philorhizus vectensis**	2
	*Porotachys bisulcatus**	1
	*Tachyta nana**	1
Cerambycidae	*Alocerus moesiacus*	6
	Cerambyx welensii	108
	Chlorophorus ruficornis	6
	*Chlorophorus trifasciatus**	2
	Phymatodes testaceus	10
	*Poecilium alni**	2
	*Prinobius myardi**	1
	*Rhagium sycophanta**	1
	*Stenopterus ater**	1
	*Stenurella nigra**	1
	*Stictoleptura fontenayi**	1
	Stictoleptura trisignata	21
	Trichoferus fasciculatus	3
	Trichoferus pallidus	19
	Xylotrechus arvicola	6
Cetoniidae	*Cetonia aurataeformis*	230
	Protaetia cuprea	97
	Protaetia mirifica	12
	Protaetia opaca	3
Ciidae	*Cis laminatus**	1
	Cis pygmaeus	13
	*Cis rugulosus**	1
	Cis striatulus	5
	*Cis vestitus**	2
	Cis villosulus	11
	Orthocis coluber	6
	Xylographus bostrichoides	9
Clambidae	*Calyptomerus dubius**	1
Cleridae	*Allonyx quadrimaculatus*	11
	Clerus mutillarius	31
	*Dermestoides sanguinicollis**	1
	*Korynetes geniculatus**	2
	*Korynetes ruficornis**	1
	*Opilo abeillei***	3
	Opilo barbarus	21
	Opilo domesticus	31
	*Thanasimus formicarius**	2

Family	Species	Individuals
	*Tilloidea unifasciata**	1
Corylophidae	*Arthrolips picea**	1
Cryptophagidae	*Atomaria atricapilla**	1
	*Atomaria pusilla**	1
	Cryptophagus aurelioi	96
	*Cryptophagus brisouti**	1
	Cryptophagus cylindrellus	5
	Cryptophagus dentatus	108
	Cryptophagus denticulatus	37
	Cryptophagus dilutus	3
	*Cryptophagus distinguendus**	2
	*Cryptophagus fallax**	1
	*Cryptophagus galantei**	1
	*Cryptophagus insulicola**	1
	Cryptophagus jakowlewi	560
	*Cryptophagus labilis**	1
	*Cryptophagus marcosgalanteorum**	1
	Cryptophagus micaceus	101
	Cryptophagus pallidus	35
	Cryptophagus pilosus	50
	*Cryptophagus populi**	2
	*Cryptophagus pseudodentatus**	1
	Cryptophagus puncticollis	10
	Cryptophagus punctipennis	52
	Cryptophagus quercinus	13
	Cryptophagus saginatus	59
	Cryptophagus scanicus	323
	*Cryptophagus spadiceus**	1
Curculionidae	*Camptorhinus simplex*	109
	Camptorhinus statua	162
	Gasterocercus hispanicus	4
	*Melicius cylindrus**	1
	*Melicius gracilis**	1
	Rhyncolus reflexus	32
	Stenoscelis submuricata	3
Curculionidae (Platypodinae)	*Platypus cylindrus*	67
Curculionidae (Scolytinae)	*Crypturgus mediterraneus**	1
	Dryocoetes villosus	238
	*Hylastes angustatus**	1

**Family**	**Species**	**Individuals**
	*Hylastinus obscurus**	1
	*Hylesinus fraxini**	1
	*Hylurgus ligniperda**	1
	*Liparthrum genistae**	1
	*Orthotomicus erosus**	1
	*Pityogenes calcaratus**	1
	*Scolytus intricatus**	2
	Xyleborinus saxesenii	279
	Xyleborus dryographus	411
	Xyleborus monographus	809
Dasytidae	*Aplocnemus albipilis**	1
	*Aplocnemus andalusicus**	1
	Aplocnemus aubei	114
	Aplocnemus brevis	32
	Aplocnemus impressus	59
	Aplocnemus limbipennis	3
	Aplocnemus nigricornis	35
	Dasytes aeratus	35
	Dasytes nigropilosus	50
	Dasytes oculatus	27
	Dasytes pauperculus	505
	Dasytes terminalis	8
	Mauroania bourgeoisi	149
	Psilothrix illustris	3
Dermestidae	*Anthrenus angustefasciatus*	26
	*Anthrenus biskrensis***	4
	*Anthrenus delicatus***	10
	Anthrenus festivus	58
	Anthrenus minutus	42
	*Anthrenus pimpinellae**	2
	*Anthrenus scrophulariae**	1
	*Anthrenus sordidulus**	1
	*Attagenus heydeni***	5
	*Attagenus schaefferi**	1
	Attagenus trifasciatus	137
	Ctesias serra	31
	Dermestes bicolor	5
	*Dermestes erichsonii**	1
	*Dermestes frischii**	2
	Dermestes hispanicus	15
	Dermestes undulatus	64

Family	Species	Individuals
	*Globicornis nigripes***	3
	*Globicornis sulcata***	119
	Orphilus niger	181
	Paranovelsis incognitus	158
	*Trogoderma inclusum***	6
	*Trogoderma versicolor**	2
Dynastidae	*Oryctes nasicornis*	8
Elateridae	*Ampedus aurilegulus*	85
	*Ampedus talamellii**	1
	*Brachygonus bouyoni**	1
	Brachygonus megerlei	5
	Brachygonus ruficeps	23
	Ectamenogonus montandoni	43
	Elater ferrugineus	78
	*Elathous rufus**	2
	Ischnodes sanguinicollis	74
	Lacon punctatus	55
	Limoniscus violaceus	9
	Megapenthes lugens	30
	*Melanotus crassicollis**	1
	Melanotus dichrous	47
	Melanotus villosus	7
	Podeonius acuticornis	18
	Procraerus tibialis	99
Endomychidae	*Mycetaea hirta**	2
	Symbiotes gibberosus	29
Erotylidae	*Triplax melanocephala**	2
Helodidae (Scirtidae)	*Prionocyphon serricornis*	129
Histeridae	*Abraeus perpusillus*	32
	Atholus corvinus	3
	*Carcinops pumilio**	2
	Dendrophilus punctatus	76
	*Gnathoncus buyssoni**	1
	Gnathoncus communis	43
	Gnathoncus nannetensis	76
	Gnathoncus rotundatus	4
	*Hetaerius ferrugineus**	1
	Kissister minimus	3
	*Margarinotus brunneus**	1
	Margarinotus merdarius	52
	*Margarinotus uncostriatus**	2

Family	Species	Individuals
	*Merohister ariasi**	2
	Paromalus filum	19
	Paromalus flavicornis	44
	*Platylomalus complanatus**	1
	*Platylomalus gardineri**	1
	*Platysoma compressum**	2
	Platysoma elongatum	5
	Platysoma filiforme	9
	*Saprinus tenuistrius**	2
Latridiidae	*Corticaria abdominalis**	1
	Corticaria inconspicua	3
	*Corticaria obscura**	2
	Enicmus brevicornis	8
	Enicmus histrio	3
	Enicmus rugosus	87
	*Enicmus transversus**	2
	*Latridius amplus**	1
	Latridius assimilis	37
	*Melanophthalma cantabrica**	1
	*Melanophthalma extensa**	1
	*Melanophthalma fuscipennis**	1
	*Melanophthlma suturalis***	3
Leiodidae	*Agathidium escorialense***	13
	*Agathidium haemorrhoum***	3
	*Agathidium ibericum**	1
	*Agathidium nigriceps**	1
	*Leiodes nigrita**	1
Lucanidae	*Dorcus parallelipipedus*	63
	*Pseudolucanus barbarossa**	2
Lymexylidae	*Lymexylon navale**	1
Malachiidae	*Anthocomus fenestratus*	20
	Axinotarsus marginalis	7
	Haplomalachius hispanus	5
	Hypebaeus albifrons	35
	*Hypebaeus alicianus**	1
	Hypebaeus flavipes	7
	*Malachius lusitanicus**	1
	*Sphinginus lobatus**	2
	Troglops furcatus	27
Melandryidae	*Abdera biflexuosa**	2
	*Conopalpus brevicollis**	1

Family	Species	Individuals
	Orchesia micans	4
	Phloiotrya tenuis	3
Melyridae	*Falsomelyris granulata*	44
Mordellidae	*Mediimorda batteni*	9
	Mordella brachyura	11
	*Mordella holomelaena**	1
	*Mordella leucaspis**	1
	Mordellistena confinis	10
	Mordellistena neuwaldeggiana	3
	Mordellochroa humerosa	3
	Tolida artemisiae	6
	*Variimorda fagniezi**	1
	*Variimorda theryi***	4
Mycetophagidae	*Eulagius filicornis**	1
	*Litargus balteatus**	1
	Litargus connexus	19
	Mycetophagus piceus	8
	Mycetophagus quadriguttatus	163
	*Typhaeola maculata**	2
Nitidulidae	*Amphotis marginata*	7
	*Amphotis martini**	1
	*Carpophilus bipustulatus**	1
	Carpophilus hemipterus	6
	Cryptarcha strigata	38
	Cryptarcha undata	22
	Epuraea fuscicollis	266
	*Epuraea ocularis**	2
	Pityophagus quercus	5
	Soronia oblonga	602
Oedemeridae	*Ischnomera xanthoderes*	134
	Oedemera flavipes	11
Prionoceridae	*Lobonyx aeneus*	25
Ptinidae (Anobiinae)	*Gastrallus immarginatus*	9
	*Gastrallus laevigatus**	2
	Hemicoelus costatus	4
	Hemicoelus nitidus	4
	Oligomerus brunneus	42
Ptinidae (Dorcatominae)	*Dorcatoma agenjoi*	21
	Dorcatoma chrysomelina	14
	*Mizodorcatoma dommeri**	1
	Stagetus andalusiacus	17

Family	Species	Individuals
	Stagetus byrrhoides	3
	*Stagetus elongatus**	1
	Stagetus micoae	4
Ptinidae (Ernobiinae)	*Xestobium rufovillosum*	15
Ptinidae (Mesocoleopodinae)	*Rhamna semen*	30
Ptinidae (Ptininae)	*Dignomus dilophus*	41
	Dignomus irroratus	128
	*Dignomus lusitanus**	1
	Ptinus bidens	749
	Ptinus hirticornis	56
	Ptinus palliatus	13
	Ptinus pyrenaeus	39
	*Ptinus sexpunctatus**	1
	Ptinus spitzyi	75
	Ptinus timidus	346
Ptinidae (Xyletininae)	*Lasioderma micros**	1
Salpingidae	*Salpingus aeneus*	27
	*Salpingus tapirus**	2
	*Sphaeriestes reyi**	1
Scraptiidae	*Anaspis flava**	2
	Anaspis humeralis	8
	*Anaspis incognita***	3
	*Anaspis kochi**	1
	Anaspis quadrimaculata	3
	Anaspis regimbarti	39
	Anaspis ruficollis	28
	*Anaspis trifasciata**	2
	Scraptia dubia	6
	Scraptia ophthalmica	3
	Scraptia testacea	112
Scydmaenidae	*Neuraphes frondosus**	2
	*Scydmaenus cornutus**	2
	*Scydmaenus perrisi**	2
	*Stenichnus collaris**	1
Silvanidae	*Ahasverus advena*	3
	*Silvanus bidentatus**	1
	Uleiota planata	7
Sphindidae	*Aspidiphorus lareyniei**	2
Tenebrionidae	*Corticeus fasciatus***	3
	Dendarus pectoralis	13
	Eledonoprius armatus	14

**Family**	**Species**	**Individuals**
	Nalassus laevioctostriatus	25
	Nephodinus villiger	7
	Palorus depressus	58
	Pentaphyllus testaceus	12
	Probaticus anthracinus	91
	Stenohelops montanus	24
	Stenohelops sublinearis	33
	Tenebrio punctipennis	117
Tenebrionidae (Alleculinae)	*Hymenalia rufipes*	9
	Isomira hispanica	148
	Mycetochara linearis	268
	Mycetochara quadrimaculata	77
	Omophlus lepturoides	4
	Prionychus ater	67
	Prionychus fairmairei	59
	Pseudocistela ceramboides	37
Tetratomidae	*Tetratoma baudueri*	28
Trogositidae	*Tenebroides maroccanus**	1
Zopheridae	*Colobicus hirtus*	5
	Colydium elongatum	27
	Endophloeus marcovichianus	46
	*Pycnomerus terebrans**	1
	*Synchita variegata**	1

Contrasting functional structure of saproxylic beetle assemblages associated to different microhabitats. Estefanía Micó, Pablo Ramilo, Simon Thorn, Jörg Müller, Eduardo Galante, Carlos P Carmona

Table 2
Species and number of individuals of saproxylic beetles from each sampling site and type of trap used for the analysis after removing rare species (singletons and doubletons) and the species with information available for less than 4 traits. Quilamas: Sierra de las Quilamas Natural Area, Cabañeros: Cabañeros National Park, Azaba: Biological Reserve "Campanarios de Azaba". ET: Hollow emergence trap, WT: window trap

**Campanarios de Azaba Biological Reserve**				
**Family**	**Species**	**ET**	**WT**	**Total**
Aderidae	*Aderus populneus*	6	4	10
	Otolelus neglectus	2	0	2
Bostrichidae	*Xylopertha praeusta*	0	4	4
Brentidae	*Amorphocephala coronata*	0	3	3
Buprestidae	*Acmaeodera degener*	0	2	2
	Acmaeoderella flavofasciata	0	5	5
	Acmaeoderella moroderi	0	4	4
	Anthaxia millefolii	0	5	5
	Chrysobothris affinis	0	3	3
Cerambycidae	*Alocerus moesiacus*	1	3	4
	Cerambyx welensii	48	26	74
	Chlorophorus ruficornis	0	3	3
	Phymatodes testaceus	0	2	2
	Trichoferus fasciculatus	0	2	2
	Trichoferus pallidus	0	5	5
	Xylotrechus arvicola	0	2	2
Cetoniidae	*Cetonia aurataeformis*	10	5	15
	Protaetia cuprea	38	8	46
	Protaetia mirifica	9	2	11
Ciidae	*Cis striatulus*	0	2	2
	Cis villosulus	1	2	3
	Xylographus bostrichoides	0	6	6
Cleridae	*Opilo domesticus*	1	5	6
Cryptophagidae	*Cryptophagus aurelioi*	30	18	48
	Cryptophagus dentatus	48	16	64
	Cryptophagus denticulatus	11	1	12
	Cryptophagus dilutus	1	2	3
	Cryptophagus jakowlewi	139	88	227
	Cryptophagus micaceus	4	0	4
	Cryptophagus pallidus	2	4	6
	Cryptophagus pilosus	41	9	50
	Cryptophagus puncticollis	0	6	6

Campanarios de Azaba Biological Reserve				
**Family**	**Species**	**ET**	**WT**	**Total**
	Cryptophagus punctipennis	1	0	1
	Cryptophagus saginatus	24	2	26
	Cryptophagus scanicus	72	41	113
Curculionidae	*Camptorhinus simplex*	0	3	3
	Camptorhinus statua	14	13	27
Curculionidae (Platypodinae)	*Platypus cylindrus*	1	17	18
Curculionidae (Scolytinae)	*Dryocoetes villosus*	0	1	1
	Xyleborinus saxesenii	6	221	227
	Xyleborus dryographus	0	11	11
	Xyleborus monographus	5	108	113
Dasytidae	*Aplocnemus brevis*	0	3	3
	Dasytes nigropilosus	0	1	1
	Dasytes oculatus	0	4	4
	Dasytes pauperculus	1	187	188
	Dasytes terminalis	0	1	1
	Mauroania bourgeoisi	1	43	44
Dermestidae	*Anthrenus angustefasciatus*	2	9	11
	Anthrenus festivus	0	7	7
	Attagenus trifasciatus	1	131	132
	Dermestes bicolor	0	4	4
	Dermestes hispanicus	0	9	9
	Dermestes undulatus	0	15	15
	Paranovelsis incognitus	3	30	33
Dynastidae	*Oryctes nasicornis*	0	1	1
Elateridae	*Ampedus aurilegulus*	0	2	2
	Brachygonus megerlei	4	0	4
	Ectamenogonus montandoni	26	15	41
	Elater ferrugineus	14	4	18
	Ischnodes sanguinicollis	2	1	3
	Lacon punctatus	6	9	15
	Limoniscus violaceus	1	2	3
	Megapenthes lugens	7	0	7
	Melanotus dichrous	0	39	39
	Procraerus tibialis	10	13	23
Endomychidae	*Symbiotes gibberosus*	5	0	5
Helodidae (Scirtidae)	*Prionocyphon serricornis*	1	0	1
Histeridae	*Abraeus perpusillus*	1	0	1
	Atholus corvinus	1	1	2

Campanarios de Azaba Biological Reserve				
**Family**	**Species**	**ET**	**WT**	**Total**
	Dendrophilus punctatus	44	29	73
	Gnathoncus communis	5	4	9
	Gnathoncus nannetensis	7	6	13
	Gnathoncus rotundatus	0	1	1
	Margarinotus merdarius	17	6	23
	Paromalus flavicornis	2	3	5
	Platysoma elongatum	0	2	2
Latridiidae	*Corticaria inconspicua*	0	2	2
	Enicmus brevicornis	0	4	4
	Enicmus rugosus	0	2	2
	Latridius assimilis	7	4	11
Lucanidae	*Dorcus parallelipipedus*	4	2	6
Malachiidae	*Anthocomus fenestratus*	3	6	9
	Axinotarsus marginalis	1	2	3
	Haplomalachius hispanus	0	2	2
	Hypebaeus albifrons	0	1	1
	Troglops furcatus	1	1	2
Melandryidae	*Phloiotrya tenuis*	0	1	1
Mordellidae	*Mediimorda batteni*	0	3	3
Mycetophagidae	*Litargus connexus*	0	6	6
	Mycetophagus quadriguttatus	39	57	96
Nitidulidae	*Amphotis marginata*	1	2	3
	Cryptarcha strigata	0	1	1
	Epuraea fuscicollis	51	41	92
	Soronia oblonga	61	157	218
Oedemeridae	*Ischnomera xanthoderes*	46	2	48
	Oedemera flavipes	0	1	1
Prionoceridae	*Lobonyx aeneus*	0	2	2
Ptinidae (Dorcatominae)	*Dorcatoma agenjoi*	0	10	10
	Stagetus byrrhoides	0	2	2
Ptinidae (Ernobiinae)	*Xestobium rufovillosum*	0	1	1
Ptinidae (Mesocoleopodinae)	*Rhamna semen*	10	4	14
Ptinidae (Ptininae)	*Ptinus bidens*	2	78	80
	Ptinus hirticornis	2	31	33
	Ptinus palliatus	0	1	1
	Ptinus pyrenaeus	2	29	31
	Ptinus spitzyi	3	36	39
	Ptinus timidus	34	70	104
Salpingidae	*Salpingus aeneus*	0	4	4

Campanarios de Azaba Biological Reserve				
**Family**	**Species**	**ET**	**WT**	**Total**
Scraptiidae	*Anaspis regimbarti*	0	3	3
	Scraptia testacea	4	0	4
Silvanidae	*Ahasverus advena*	0	1	1
Tenebrionidae	*Nephodinus villiger*	0	7	7
	Palorus depressus	5	30	35
	Probaticus anthracinus	42	8	50
	Tenebrio punctipennis	18	53	71
Tenebrionidae (Alleculinae)	*Hymenalia rufipes*	0	6	6
	Isomira hispanica	0	14	14
	Mycetochara linearis	3	75	78
	Mycetochara quadrimaculata	3	22	25
	Omophlus lepturoides	0	2	2
	Prionychus ater	13	23	36
	Prionychus fairmairei	0	2	2
Tetratomidae	*Tetratoma baudueri*	0	7	7
Zopheridae	*Colydium elongatum*	0	4	4
**Total**		**1031**	**2068**	**3099**

Cabañeros National Park				
**Family**	**Species**	**ET**	**WT**	**Total**
Aderidae	*Aderus populneus*	26	0	26
Biphyllidae	*Diplocoelus fagi*	8	5	13
Bothrideridae	*Bothrideres interstitialis*	0	2	2
	Oxylaemus cylindricus	0	3	3
Brentidae	*Amorphocephala coronata*	0	3	3
Buprestidae	*Acmaeodera degener*	0	2	2
	Anthaxia millefolii	0	1	1
	Anthaxia salicis	0	3	3
Carabidae	*Dromius agilis*	0	1	1
Cerambycidae	*Alocerus moesiacus*	2	0	2
	Cerambyx welensii	3	2	5
	Phymatodes testaceus	0	6	6
	Stictoleptura trisignata	20	0	20
	Trichoferus fasciculatus	1	0	1
	Trichoferus pallidus	0	3	3
	Xylotrechus arvicola	0	1	1
Cetoniidae	*Cetonia aurataeformis*	144	2	146
	Protaetia cuprea	6	0	6

Family	Species	ET	WT	Total
	Protaetia opaca	1	1	2
Ciidae	*Cis striatulus*	0	1	1
	Cis villosulus	0	1	1
Cleridae	*Opilo domesticus*	1	18	19
Cryptophagidae	*Cryptophagus aurelioi*	14	1	15
	Cryptophagus cylindrellus	4	0	4
	Cryptophagus dentatus	11	17	28
	Cryptophagus jakowlewi	96	0	96
	Cryptophagus micaceus	73	7	80
	Cryptophagus pallidus	0	29	29
	Cryptophagus punctipennis	22	0	22
	Cryptophagus saginatus	24	2	26
	Cryptophagus scanicus	88	9	97
Curculionidae	*Camptorhinus simplex*	2	6	8
	Camptorhinus statua	89	7	96
	Gasterocercus hispanicus	2	2	4
	Stenoscelis submuricata	0	3	3
Curculionidae (Platypodinae)	*Platypus cylindrus*	0	2	2
Curculionidae (Scolytinae)	*Dryocoetes villosus*	0	1	1
	Xyleborinus saxesenii	11	11	22
	Xyleborus dryographus	6	27	33
	Xyleborus monographus	165	113	278
Dasytidae	*Aplocnemus brevis*	0	7	7
	Aplocnemus limbipennis	0	3	3
	Dasytes pauperculus	0	74	74
	Mauroania bourgeoisi	1	90	91
	Psilothrix illustris	0	3	3
Dermestidae	*Anthrenus angustefasciatus*	0	6	6
	Anthrenus festivus	1	44	45
	Anthrenus minutus	0	1	1
	Attagenus trifasciatus	1	4	5
	Dermestes bicolor	1	0	1
	Dermestes undulatus	5	0	5
	Orphilus niger	2	8	10
	Paranovelsis incognitus	1	23	24
Dynastidae	*Oryctes nasicornis*	7	0	7
Elateridae	*Ampedus aurilegulus*	28	12	40

Family	Species	ET	WT	Total
	Ectamenogonus montandoni	2	0	2
	Elater ferrugineus	25	0	25
	Ischnodes sanguinicollis	21	9	30
	Lacon punctatus	4	7	11
	Megapenthes lugens	15	1	16
	Podeonius acuticornis	0	2	2
	Procraerus tibialis	2	2	4
Endomychidae	*Symbiotes gibberosus*	2	3	5
Helodidae (Scirtidae)	*Prionocyphon serricornis*	122	0	122
Histeridae	*Abraeus perpusillus*	24	1	25
	Gnathoncus communis	34	0	34
	Gnathoncus nannetensis	10	10	20
	Gnathoncus rotundatus	0	3	3
	Kissister minimus	0	3	3
	Margarinotus merdarius	12	2	14
	Paromalus flavicornis	23	1	24
	Platysoma filiforme	1	0	1
Latridiidae	*Enicmus brevicornis*	3	0	3
	Enicmus rugosus	4	0	4
	Latridius assimilis	14	1	15
Lucanidae	*Dorcus parallelipipedus*	34	5	39
Malachiidae	*Anthocomus fenestratus*	11	0	11
	Hypebaeus albifrons	3	0	3
	Troglops furcatus	25	0	25
Melandryidae	*Orchesia micans*	4	0	4
Melyridae	*Falsomelyris granulata*	0	43	43
Mordellidae	*Mediimorda batteni*	0	6	6
	Mordella brachyura	0	8	8
Mycetophagidae	*Litargus connexus*	9	3	12
	Mycetophagus quadriguttatus	51	2	53
Nitidulidae	*Amphotis marginata*	2	2	4
	Epuraea fuscicollis	127	8	135
	Soronia oblonga	136	116	252
Oedemeridae	*Ischnomera xanthoderes*	49	0	49
Prionoceridae	*Lobonyx aeneus*	0	3	3
Ptinidae (Anobiinae)	*Oligomerus brunneus*	20	17	37
Ptinidae (Dorcatominae)	*Dorcatoma agenjoi*	8	0	8
	Stagetus andalusiacus	0	17	17
Ptinidae (Mesocoleopodinae)	*Rhamna semen*	13	2	15

**Family**	**Species**	**ET**	**WT**	**Total**
Ptinidae (Ptininae)	*Dignomus dilophus*	0	41	41
	Dignomus irroratus	3	125	128
	Ptinus bidens	10	10	20
	Ptinus hirticornis	1	18	19
	Ptinus palliatus	0	11	11
	Ptinus pyrenaeus	0	2	2
	Ptinus spitzyi	1	25	26
	Ptinus timidus	174	13	187
Salpingidae	*Salpingus aeneus*	0	11	11
Scraptiidae	*Anaspis humeralis*	0	2	2
	Anaspis regimbarti	10	1	11
	Scraptia dubia	0	5	5
	Scraptia ophthalmica	0	3	3
	Scraptia testacea	25	0	25
Silvanidae	*Ahasverus advena*	1	0	1
	Uleiota planata	5	0	5
Tenebrionidae	*Eledonoprius armatus*	7	1	8
	Pentaphyllus testaceus	0	1	1
	Probaticus anthracinus	34	7	41
	Stenohelops sublinearis	3	30	33
	Tenebrio punctipennis	29	4	33
Tenebrionidae (Alleculinae)	*Isomira hispanica*	0	54	54
	Mycetochara linearis	4	12	16
	Mycetochara quadrimaculata	21	31	52
	Prionychus fairmairei	8	13	21
	Pseudocistela ceramboides	36	1	37
Tetratomidae	*Tetratoma baudueri*	0	5	5
Zopheridae	*Colobicus hirtus*	1	4	5
	Colydium elongatum	5	8	13
	Endophloeus marcovichianus	22	20	42
**Total**		**2076**	**1302**	**3378**

Sierra de las Quilamas Natural Area

Family	Species	ET	WT	Total
Aderidae	*Otolelus neglectus*	1	6	7
Anthribidae	*Allandrus therondi*	0	3	3
Bostrichidae	*Xylopertha praeusta*	0	1	1
Bothrideridae	*Bothrideres interstitialis*	0	4	4

Family	Species	ET	WT	Total
	Oxylaemus cylindricus	0	10	10
Brentidae	*Amorphocephala coronata*	0	19	19
Buprestidae	*Acmaeodera degener*	0	6	6
	Acmaeoderella adspersula	0	9	9
	Anthaxia carmen	0	9	9
	Anthaxia millefolii	0	10	10
	Anthaxia salicis	0	2	2
	Chrysobothris affinis	0	33	33
Cantharidae	*Malthodes forcipipher*	0	16	16
Carabidae	*Dromius agilis*	0	5	5
	Lamprias rufipes	0	10	10
	Lebia marginata	0	3	3
	Metadromius myrmidon	0	3	3
Cerambycidae	*Cerambyx welensii*	23	6	29
	Chlorophorus ruficornis	0	3	3
	Phymatodes testaceus	0	2	2
	Stictoleptura trisignata	0	1	1
	Trichoferus pallidus	0	11	11
	Xylotrechus arvicola	0	3	3
Cetoniidae	*Cetonia aurataeformis*	54	15	69
	Protaetia cuprea	19	26	45
	Protaetia mirifica	0	1	1
	Protaetia opaca	0	1	1
Ciidae	*Cis pygmaeus*	0	13	13
	Cis striatulus	0	2	2
	Cis villosulus	0	7	7
	Orthocis coluber	0	6	6
	Xylographus bostrichoides	0	3	3
Cleridae	*Allonyx quadrimaculatus*	0	11	11
	Clerus mutillarius	0	31	31
	Opilo barbarus	0	21	21
	Opilo domesticus	0	6	6
Cryptophagidae	*Cryptophagus aurelioi*	8	25	33
	Cryptophagus cylindrellus	0	1	1
	Cryptophagus dentatus	7	9	16
	Cryptophagus denticulatus	1	24	25

Family	Species	ET	WT	Total
	Cryptophagus jakowlewi	71	166	237
	Cryptophagus micaceus	0	17	17
	Cryptophagus puncticollis	0	4	4
	Cryptophagus punctipennis	21	8	29
	Cryptophagus quercinus	0	13	13
	Cryptophagus saginatus	2	5	7
	Cryptophagus scanicus	15	98	113
Curculionidae	*Camptorhinus simplex*	8	90	98
	Camptorhinus statua	27	12	39
	Rhyncolus reflexus	6	26	32
Curculionidae (Platypodinae)	*Platypus cylindrus*	0	47	47
Curculionidae (Scolytinae)	*Dryocoetes villosus*	0	236	236
	Xyleborinus saxesenii	2	28	30
	Xyleborus dryographus	3	364	367
	Xyleborus monographus	5	413	418
Dasytidae	*Aplocnemus aubei*	0	114	114
	Aplocnemus brevis	0	22	22
	Aplocnemus impressus	0	59	59
	Aplocnemus nigricornis	1	34	35
	Dasytes aeratus	0	35	35
	Dasytes nigropilosus	0	49	49
	Dasytes oculatus	0	23	23
	Dasytes pauperculus	0	243	243
	Dasytes terminalis	0	7	7
	Mauroania bourgeoisi	0	14	14
Dermestidae	*Anthrenus angustefasciatus*	0	9	9
	Anthrenus festivus	0	6	6
	Anthrenus minutus	5	36	41
	Ctesias serra	4	27	31
	Dermestes hispanicus	1	5	6
	Dermestes undulatus	1	43	44
	Orphilus niger	2	169	171
	Paranovelsis incognitus	2	99	101

Family	Species	ET	WT	Total
Elateridae	*Ampedus aurilegulus*	4	39	43
	Brachygonus megerlei	0	1	1
	Brachygonus ruficeps	0	23	23
	Elater ferrugineus	18	17	35
	Ischnodes sanguinicollis	18	23	41
	Lacon punctatus	4	25	29
	Limoniscus violaceus	4	2	6
	Megapenthes lugens	4	3	7
	Melanotus dichrous	4	4	8
	Melanotus villosus	2	5	7
	Podeonius acuticornis	0	16	16
	Procraerus tibialis	10	62	72
Endomychidae	*Symbiotes gibberosus*	2	17	19
Helodidae (Scirtidae)	*Prionocyphon serricornis*	4	2	6
Histeridae	*Abraeus perpusillus*	3	3	6
	Atholus corvinus	0	1	1
	Dendrophilus punctatus	1	2	3
	Gnathoncus nannetensis	2	41	43
	Margarinotus merdarius	3	12	15
	Paromalus filum	0	19	19
	Paromalus flavicornis	3	12	15
	Platysoma elongatum	0	3	3
	Platysoma filiforme	0	8	8
Latridiidae	*Corticaria inconspicua*	1	0	1
	Enicmus brevicornis	0	1	1
	Enicmus histrio	0	3	3
	Enicmus rugosus	10	71	81
	Latridius assimilis	8	3	11
Lucanidae	*Dorcus parallelipipedus*	12	6	18
Malachiidae	*Axinotarsus marginalis*	1	3	4
	Haplomalachius hispanus	0	3	3
	Hypebaeus albifrons	13	18	31
	Hypebaeus flavipes	6	1	7
Melandryidae	*Phloiotrya tenuis*	0	2	2
Melyridae	*Falsomelyris granulata*	0	1	1
Mordellidae	*Mordella brachyura*	0	3	3
	Mordellistena confinis	0	10	10

Family	Species	ET	WT	Total
	Mordellistena neuwaldeggiana	0	3	3
	Mordellochroa humerosa	0	3	3
	Tolida artemisiae	0	6	6
Mycetophagidae	*Litargus connexus*	0	1	1
	Mycetophagus piceus	0	8	8
	Mycetophagus quadriguttatus	4	10	14
Nitidulidae	*Carpophilus hemipterus*	0	6	6
	Cryptarcha strigata	0	37	37
	Cryptarcha undata	0	22	22
	Epuraea fuscicollis	5	34	39
	Pityophagus quercus	0	5	5
	Soronia oblonga	7	125	132
Oedemeridae	*Ischnomera xanthoderes*	30	7	37
	Oedemera flavipes	0	10	10
Prionoceridae	*Lobonyx aeneus*	1	19	20
Ptinidae (Anobiinae)	*Gastrallus immarginatus*	0	9	9
	Hemicoelus costatus	0	4	4
	Hemicoelus nitidus	2	2	4
	Oligomerus brunneus	1	4	5
Ptinidae (Dorcatominae)	*Dorcatoma agenjoi*	0	3	3
	Dorcatoma chrysomelina	2	12	14
	Stagetus byrrhoides	0	1	1
	Stagetus micoae	0	4	4
Ptinidae (Ernobiinae)	*Xestobium rufovillosum*	3	11	14
Ptinidae (Mesocoleopodinae)	*Rhamna semen*	0	1	1
Ptinidae (Ptininae)	*Ptinus bidens*	107	542	649
	Ptinus hirticornis	1	3	4
	Ptinus palliatus	0	1	1
	Ptinus pyrenaeus	0	6	6
	Ptinus spitzyi	0	10	10
	Ptinus timidus	19	36	55
Salpingidae	*Salpingus aeneus*	0	12	12
Scraptiidae	*Anaspis humeralis*	0	6	6
	Anaspis quadrimaculata	0	3	3

Family	Species	ET	WT	Total
	Anaspis regimbarti	0	25	25
	Anaspis ruficollis	5	23	28
	Scraptia dubia	0	1	1
	Scraptia testacea	62	21	83
Silvanidae	*Ahasverus advena*	0	1	1
	Uleiota planata	0	2	2
Tenebrionidae	*Dendarus pectoralis*	13	0	13
	Eledonoprius armatus	0	6	6
	Nalassus laevioctostriatus	6	19	25
	Palorus depressus	3	20	23
	Pentaphyllus testaceus	0	11	11
	Stenohelops montanus	2	22	24
	Tenebrio punctipennis	4	9	13
Tenebrionidae (Alleculinae)	*Hymenalia rufipes*	0	3	3
	Isomira hispanica	3	77	80
	Mycetochara linearis	11	163	174
	Omophlus lepturoides	0	2	2
	Prionychus ater	14	17	31
	Prionychus fairmairei	3	33	36
Tetratomidae	*Tetratoma baudueri*	0	16	16
Zopheridae	*Colydium elongatum*	0	10	10
	Endophloeus marcovichianus	0	4	4
**Total**		**734**	**4814**	**5548**

Contrasting functional structure of saproxylic beetle assemblages associated to different microhabitats. Estefanía Micó, Pablo Ramilo, Simon Thorn, Jörg Müller, Eduardo Galante, Carlos P Carmona

Species richness and diversity using the whole matrix

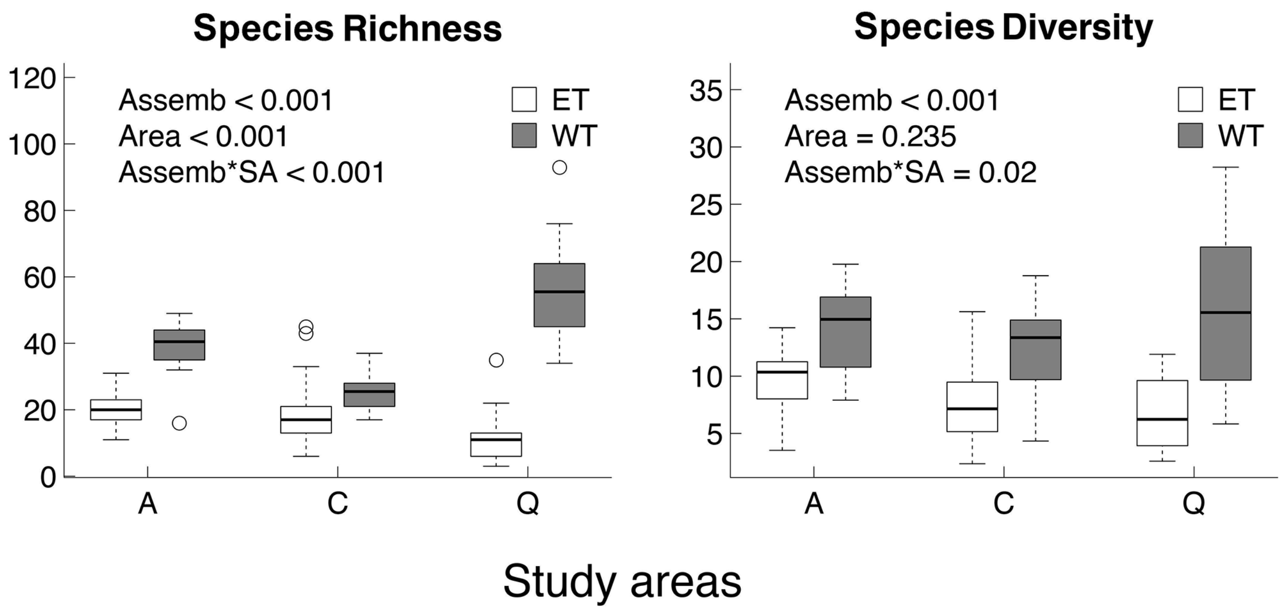

Effects of the kind of assemblage (Assemb; hollow assemblage (ET) (in white) or collected by window trap (WT) (in gray)), the study area (SA; A: Azaba, C: Cabañeros, Q: Quilamas) and its interaction on the species richness and diversity using the whole matrix (without removing singletons and doubletons)

Contrasting functional structure of saproxylic beetle assemblages associated to different microhabitats. Estefanía Micó, Pablo Ramilo, Simon Thorn, Jörg Müller, Eduardo Galante, Carlos P Carmona

Location of study sites and traps

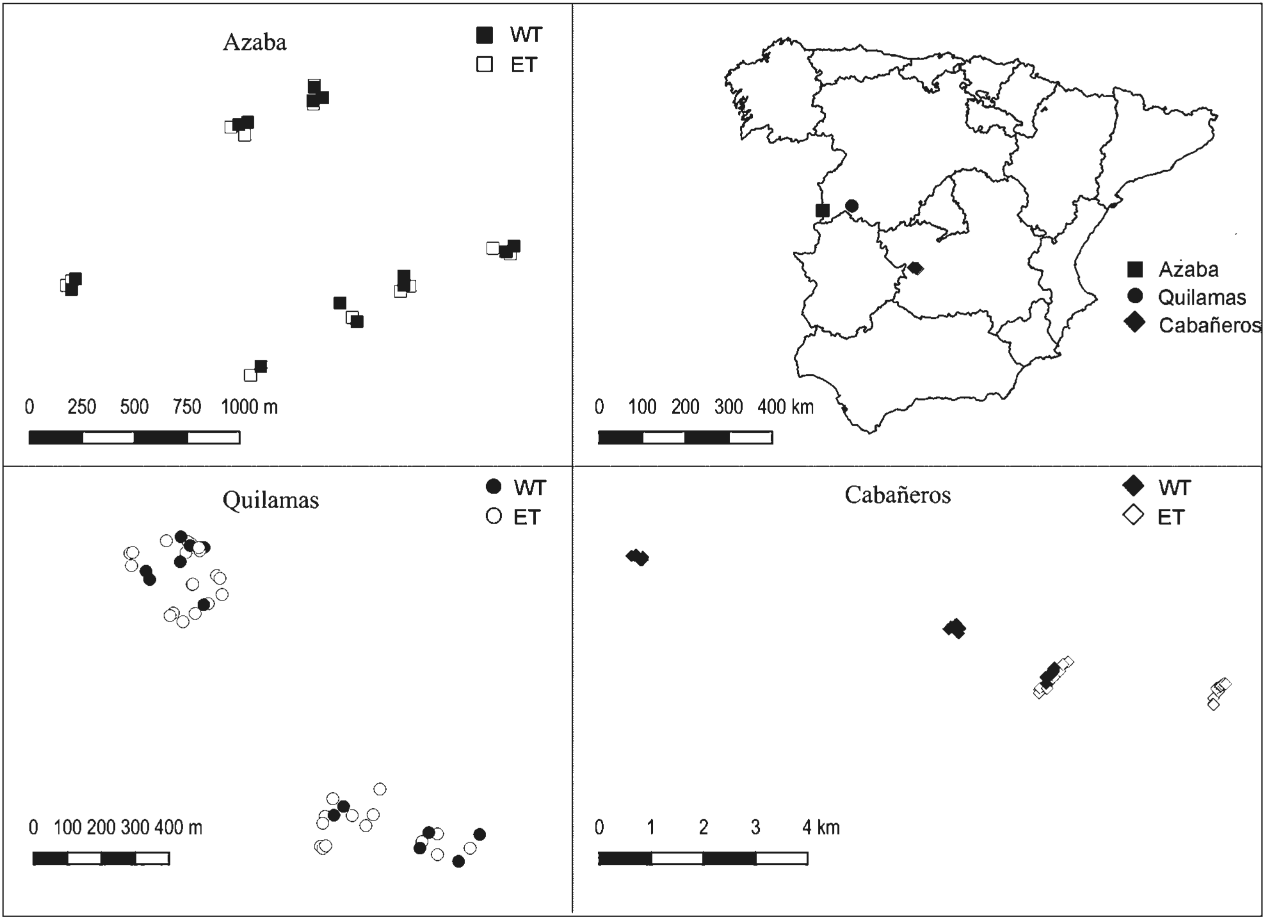

Supplement: Supplementary file 1 — Supplementary Table S1 and S2 and Figure S3 and S4. [file 41598_2020_58408_MOESM1_ESM.pdf]
